# The Role of Systemic Inflammation in Paracetamol Poisoning—An Experimental Model

**DOI:** 10.3390/ijms27177891

**Published:** 2026-09-04

**Authors:** Alevtina Zudova, Svetlana Apanovich, Elena Mukhlynina, Liliya Solomatina, Evgenii Gusev

**Affiliations:** 1 Institute of Immunology and Physiology, Ural Branch of the Russian Academy of Sciences, 620078 Yekaterinburg, Russia; tina.zudova@mail.ru (A.Z.); elena.mukhlynina@yandex.ru (E.M.); gusev36@mail.ru (E.G.); 2 Institute of Fundamental Medicine, Ural State Medical University, 620028 Yekaterinburg, Russia; 3 Institute of Natural Sciences and Mathematics, Ural Federal University, 620062 Yekaterinburg, Russia; apanovich.lanochka@yandex.ru

**Keywords:** acetaminophen, multiple organ failure, cytokine storm, systemic inflammation, experimental model

## Abstract

Accumulating evidence indicates that acute, life-threatening acetaminophen (APAP) poisoning is associated with systemic inflammation (SI). Investigating SI in experimental models holds promise for the development of pathogenetic therapies. This study aimed to evaluate SI manifestations in a murine model of APAP-induced toxic liver injury. Ten-week-old male C57Bl/6 mice received a single intraperitoneal dose of 600 mg/kg APAP, corresponding to the median lethal dose (LD50). To assess signs of multiorgan damage and SI, we performed histological examinations of internal organs as well as immunoenzymatic, hematological, and biochemical analyses of blood samples. Histological analysis revealed tissue alterations in the liver, kidneys, spleen, lungs, and heart, accompanied by functional impairment. Statistically significant changes were observed in plasma levels of tumor necrosis factor-α (TNF-α), IL-1α, IL-6, IL-10, C-reactive protein (CRP), platelet count, total bilirubin, alanine aminotransferase (ALT), aspartate aminotransferase (AST), creatinine, and troponin I. Severe liver damage induced by APAP was associated with a cytokine storm, coagulopathy (thrombocytopenia), systemic alterations, pulmonary edema, and multiorgan dysfunction. Collectively, these findings indicate that severe APAP-induced liver damage triggers SI as a characteristic pathological process.

## 1. Introduction

Acetaminophen (paracetamol, N-acetyl-para-aminophenol, APAP) is a widely used analgesic and antipyretic agent available over the counter and by prescription. Its widespread availability contributes to frequent unintentional or intentional overdoses, which can lead to acute hepatotoxicity and substantial mortality [1,2,3].

At supratherapeutic doses, APAP metabolism saturates the sulfation pathway due to depletion of its cofactor, 3′-phosphoadenosine-5′-phosphosulfate (PAPS), shifting metabolism toward glucuronidation and cytochrome P450-mediated oxidation. This shift promotes excessive formation of the highly reactive intermediate N-acetyl-p-benzoquinone imine (NAPQI). Current evidence indicates that NAPQI accumulation in acute APAP overdose initiates a multifaceted hepatotoxic cascade, characterized by: (i) depletion of intracellular glutathione stores [4,5]; (ii) overproduction of reactive oxygen species (ROS) [6,7]; and (iii) disruption of mitochondrial and endoplasmic reticulum homeostasis within hepatocytes [7,8,9]. Collectively, these events trigger widespread hepatocellular necrosis, coupled with the release of damage-associated molecular patterns (DAMPs) into the extracellular space. DAMPs act as endogenous danger signals, activating innate immune cells and orchestrating the secretion of a broad spectrum of pro- and anti-inflammatory cytokines.

Although APAP metabolism is well understood, the mechanisms of the associated inflammatory response are a matter of ongoing debate [10,11]. Specifically, the significance of sterile inflammation in APAP hepatotoxicity remains unclear—whether it exacerbates injury at later stages or serves solely to remove necrotic material and promote regenerative repair. We suggest that the amplified inflammatory response originating in the liver may spread systemically and eventually lead to SI.

SI is a general pathological process that arises from systemic injury and is marked by the inflammatory reactivity of endothelial cells, blood components, and connective tissue, progressing, in its final stages, to microcirculatory disturbances in vital organs and tissues [12].

SI encompasses several interconnected phenomena that are causally linked [12,13]:

1. Systemic inflammatory response (SIR)—accumulation of high concentrations of inflammatory mediators and other cellular stress products in the circulation in response to systemic damaging factors.
2. Systemic alteration—characterized by dissemination of tissue damage markers in the bloodstream and/or morphological evidence of multiorgan injury.
3. Microcirculatory disorders (MCD)—a key pathogenetic link in the development of various shock states, most cases of multiple organ failure (MOF), and disseminated intravascular coagulation (DIC).
4. MOF—assessed using the Sequential Organ Failure Assessment (SOFA) score [14], which is the widely accepted standard in critical care. However, no such standardized criteria exist for experimental animals, complicating the establishment of species-specific MOF and DIC criteria.
5. Neuroendocrine dysfunction—an important component of SI, reflected by signs of the “distress” variant of the general adaptation syndrome, notably dysfunction of the hypothalamic–pituitary–adrenal (HPA) axis.

To assess the intensity of the immune response and detect SI in patients, the Laboratory of Inflammation Immunology at the Institute of Immunology and Physiology of the Ural Branch of the Russian Academy of Sciences has developed an integrated SI scale. This scoring system includes the following parameters: CRP and a panel of cytokines (IL-1β, IL-6, IL-8, IL-10, and TNF-α) for the SIR; D-dimer for DIC; cortisol for HPA axis distress; troponin I and myoglobin for systemic alteration; and the SOFA score for MOF [15]. The latter additionally incorporates platelet count, bilirubin, and creatinine [14].

Evidence indicates that patients with acute liver failure (ALF), including APAP-induced cases, exhibit various SI phenomena, such as coagulopathy, MOF [16,17,18,19], and SIR [20,21,22,23]. The role of specific cytokines in APAP overdose has received considerable attention. It is well established that IL-1α acts as both an alarmin and a cytokine [10,24], and the kinetics of IL-6, TNF-α, IL-10, and other soluble mediators have been characterized in rodents and humans, with some of these emerging as potential markers of poor outcome [23,25,26]. Nevertheless, the inflammatory pathogenesis in APAP overdose remains a matter of ongoing controversy. Moreover, a comprehensive evaluation of systemic inflammatory manifestations in life-threatening APAP intoxication has not been previously undertaken.

The potential for developing SI is rooted in the evolutionary complexity of the immune, hemostatic, and microcirculatory systems—a trait shared by humans and many mammals, including rodents [27]. Importantly, the key mechanisms of severe APAP-induced liver injury are similar in humans and mice [28]. Thus, the mouse model of APAP-induced ALF closely mirrors the human condition, allowing more detailed study of organ pathology and immune responses over time. Studying SI in this model can deepen our understanding of APAP hepatotoxicity and may help identify new therapeutic targets.

Thus, this study aimed to evaluate SI in a mouse model of APAP-induced hepatotoxicity.

## 2. Results

### 2.1. Systemic Physiological Changes and Clinical Severity

**Figure 1:**
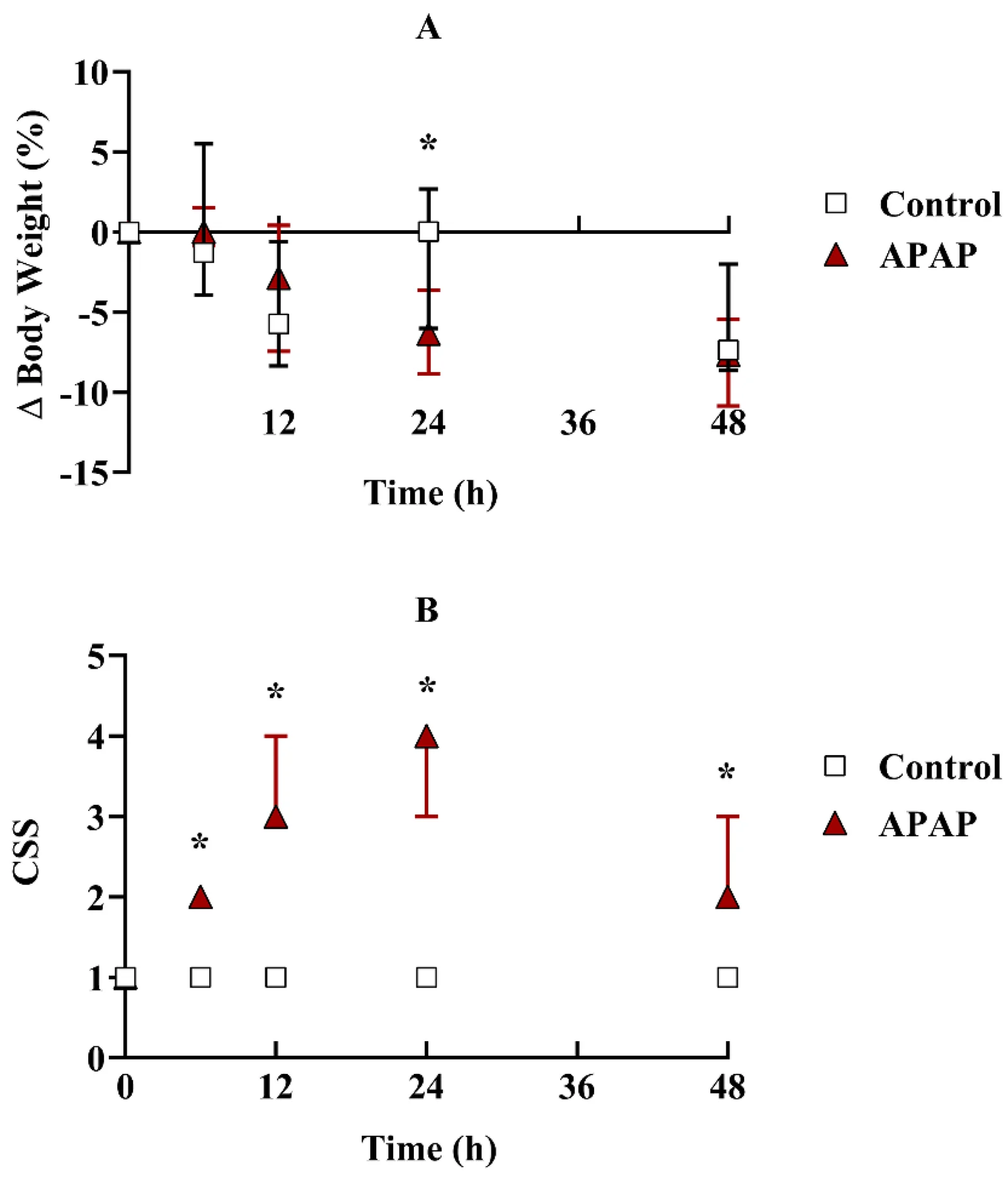
Effect of LD50 acetaminophen (APAP) administration on body weight gain and clinical severity score in C57Bl/6 mice. (**A**) Body weight gain. (**B**) Clinical severity score. Data are presented as medians with interquartile range. N = 7 mice per group. Mann–Whitney U-test, *—*p* < 0.05.

APAP-treated mice exhibited a pronounced decline in overall status, characterized by hypothermia, bradypnea, diminished food and water consumption, and oliguria. Notably, body weight was significantly lower than in the controls at the 24 h time point (Figure 1A).

Clinical severity was assessed using the clinical severity score (CSS) [29] (Figure 1B). Control and intact animals uniformly scored 1.00, indicating normal behavior. In contrast, APAP-treated mice showed a significant increase in CSS at all time points from 6 h to 48 h compared with controls. The scores increased over time, peaking at 24 h, and correlated with progressive clinical deterioration, including reduced mobility, hunched posture, blunted responsiveness, and—at the maximum—pronounced piloerection, lethargy, and reluctance to move upon gentle provocation. No deaths occurred before 12 h; the highest mortality was observed at 24 h, yielding an overall survival rate of 70% at 48 h.

### 2.2. Hematological Parameters

**Figure 2:**
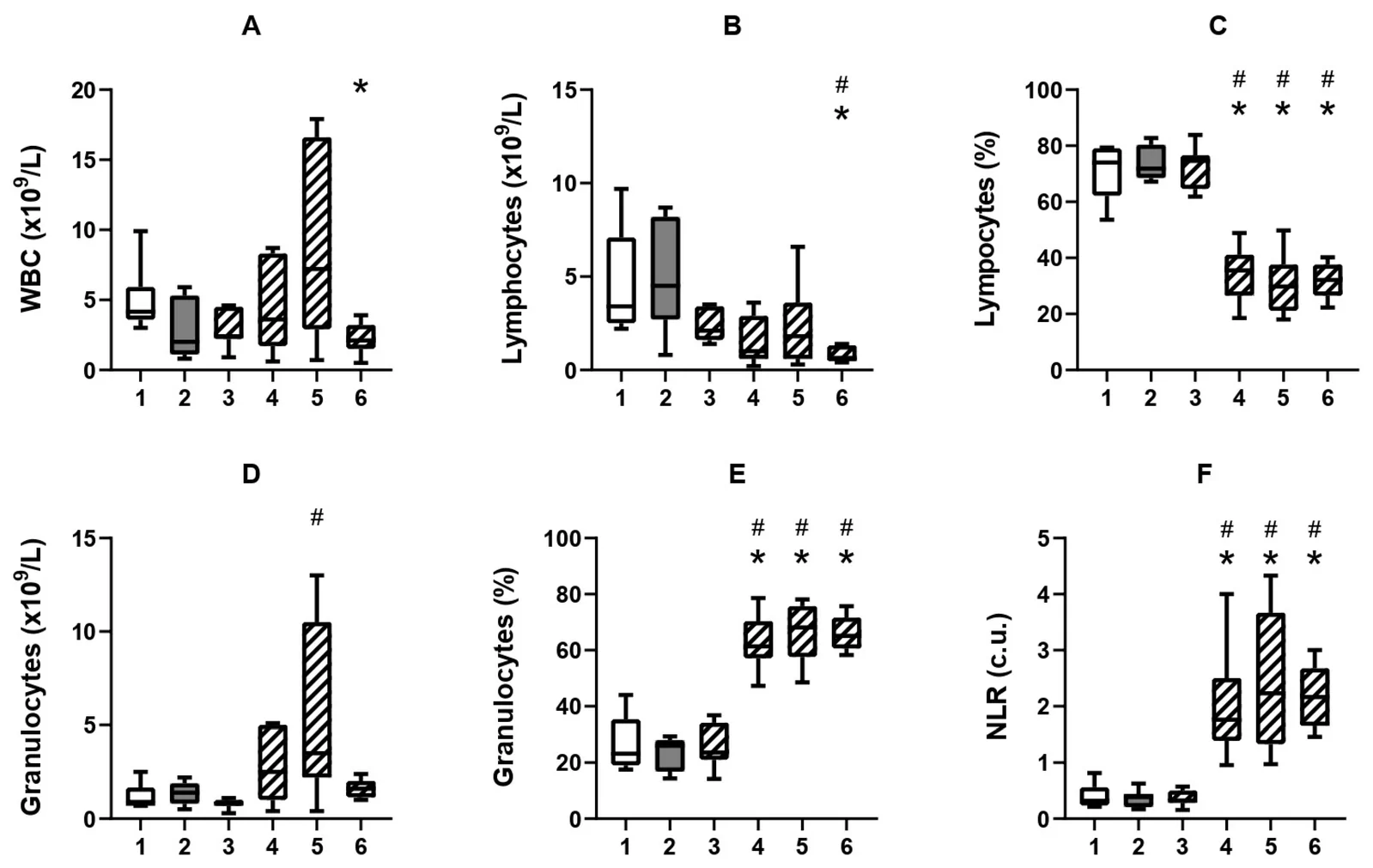
The effects of LD50 acetaminophen (APAP) administration on hematological parameters in C57Bl/6 mice. (**A**) White blood cells (WBCs). (**B**) Absolute lymphocyte count. (**C**) Lymphocyte percentage. (**D**) Absolute granulocyte count. (**E**) Granulocyte percentage. (**F**) Neutrophil-to-lymphocyte ratio (NLR). 1—intact group. 2—control group. 3—APAP, 6 h. 4—APAP, 12 h. 5—APAP, 24 h. 6—APAP, 48 h. The data are presented in box and whisker plots: the boxes are drawn from Q1 to Q3, the bold lines show medians, and the whiskers provide the 100% ranges of the values. N = 7 mice per group. Mann–Whitney U-test, *—*p* < 0.05 vs. intact group; #—*p* < 0.05 vs. control group.

In APAP-treated mice, the absolute white blood cell (WBC) count remained largely stable over most time points, with a significant drop only at 48 h versus intact controls (Figure 2A). Absolute lymphocyte counts fell significantly only at 48 h post-APAP (Figure 2B), whereas relative lymphopenia was already evident at 12 h and persisted thereafter (Figure 2C). Relative granulocytosis began at 12 h and lasted through 48 h (Figure 2E); however, the absolute granulocyte count was significantly elevated only at 24 h (Figure 2D). The neutrophil-to-lymphocyte ratio (NLR) increased significantly from 12 h to 48 h (Figure 2F). In patients with APAP overdose, a similar elevation in NLR has been linked to poor outcomes, SI, and hepatic encephalopathy [30].

### 2.3. Systemic Inflammatory Response

**Figure 3:**
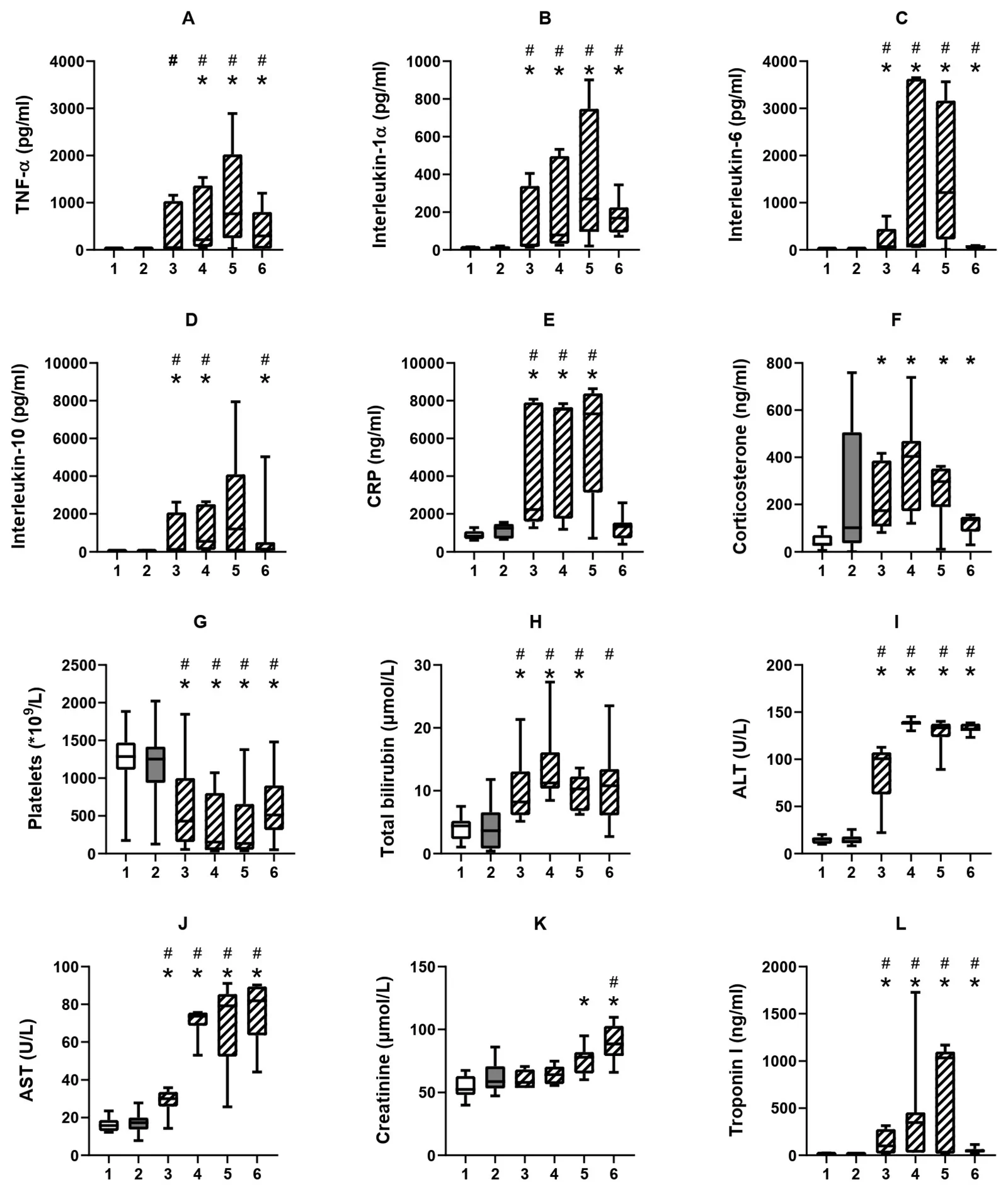
The effects of LD50 acetaminophen (APAP) administration on systemic inflammation (SI) phenomena in C57Bl/6 mice. (**A**) Tumor necrosis factor-α (TNF-α). (**B**) Interleukin-1α. (**C**) Interleukin-6. (**D**) Interleukin-10. (**E**) C-reactive protein (CRP). (**F**) Corticosterone. (**G**) Platelets. (**H**) Total bilirubin. (**I**) Alanine aminotransferase (ALT). (**J**) Aspartate aminotransferase (AST). (**K**) Creatinine. (**L**) Troponin-I. 1—intact group. 2—control group. 3—APAP, 6 h. 4—APAP, 12 h. 5—APAP, 24 h. 6—APAP, 48 h. The data are presented in box and whisker plots: the boxes are drawn from Q1 to Q3, the bold lines show medians, and the whiskers provide the 100% ranges of the values. N = 7 mice per group. Mann–Whitney U-test, *—*p* < 0.05 vs. intact group; #—*p* < 0.05 vs. control group.

Following the administration of a sublethal dose of APAP, C57Bl/6 mice developed a generalized SIR, characterized by a significant increase in the plasma levels of pro-inflammatory cytokines (IL-1α, IL-6, and TNF-α), the anti-inflammatory cytokine IL-10, and CRP relative to the control animals at all studied time points. The concentrations of all markers reached their maxima at 24 h post-injection (Figure 3A–E). At this time point, median cytokine levels in the experimental groups were significantly elevated compared with the controls: TNF-α (39-fold), IL-1α (25-fold), IL-6 (135-fold), IL-10 (45-fold), and CRP (6-fold). These findings are consistent with a cytokine storm, and the substantial CRP increase further confirms an acute-phase reaction.

### 2.4. Activation of the Hypothalamic–Pituitary–Adrenal Axis

To assess whether APAP administration elicited a distress response via the HPA axis, plasma corticosterone levels were measured. A significant increase in corticosterone was observed in the experimental groups compared with the intact animals (Figure 3F). However, corticosterone concentrations in the control group showed considerable variability, and a significant difference was also observed between the intact and control animals.

### 2.5. Assessment of Paracoagulation

APAP-induced hepatotoxicity was associated with the development of intravascular microthrombosis, as reflected by a significant decrease in absolute platelet counts in the experimental groups relative to controls (Figure 3G).

### 2.6. Multiple Organ Alteration and Dysfunction

Histological examination revealed that the organs of the intact and control groups exhibited normal tissue architecture.

#### 2.6.1. Histological and Functional Characteristics of the Liver

**Figure 4:**
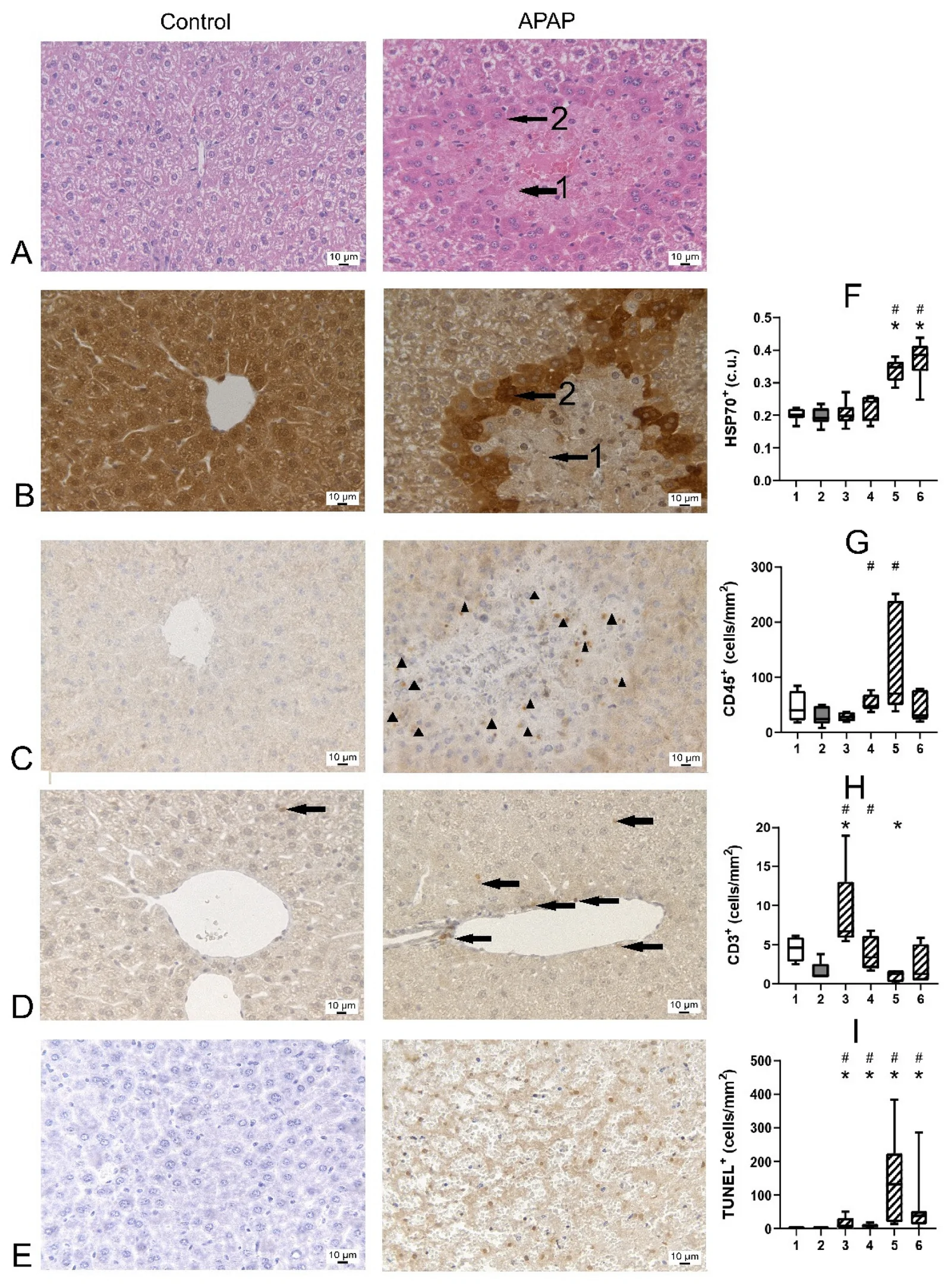
The effect of LD50 acetaminophen (APAP) administration on the liver histology in C57Bl/6 mice. (**A**) Representative microphotographs of the liver sections stained by hematoxylin and eosin (H&E). APAP—24 h. 1—necrotic zone; 2—inflammatory demarcation zone. (**B**) Representative microphotographs of HSP70 stained liver sections. APAP—24 h. 1—necrotic zone; 2—inflammatory demarcation zone. (**C**) Representative microphotographs of CD45 stained liver sections. APAP—24 h. Black triangles denote CD45^+^-cells. (**D**) Representative microphotographs of CD3 stained liver sections. APAP—6 h. Black arrows denote CD3^+^-cells. (**E**) Representative microphotographs of TUNEL stained liver sections. APAP—24 h. Objective magnification 40×. Scale bar—10 µm. (**F**) Optical density of HSP70^+^-cells. (**G**) Liver CD45^+^-cells. (**H**) Liver CD3^+^-cells. (**I**) Liver TUNEL^+^-cells. 1—intact group. 2—control group. 3—APAP, 6 h. 4—APAP, 12 h. 5—APAP, 24 h. 6—APAP, 48 h. The data are presented in box and whisker plots: the boxes are drawn from Q1 to Q3, the bold lines show medians, and the whiskers provide the 100% ranges of the values. N = 7 mice per group. Mann–Whitney U-test, *—*p* < 0.05 vs. intact group; #—*p* < 0.05 vs. control group.

Six hours following APAP administration, centrilobular hemorrhagic infiltration of the hepatic parenchyma was observed, alongside distinct signs of hepatocellular injury. These dystrophic changes were characterized by cellular and nuclear pleomorphism (anisocytosis and anisonucleosis), heterogeneous staining patterns, cytoplasmic vacuolization, and cellular enlargement. By 12 h post-administration, destructive changes had intensified, manifesting as the development of centrilobular hepatocyte necrosis. At the 24-h time point, a notable expansion of necrotic foci was observed, accompanied by immune cell infiltration and the establishment of an inflammatory demarcation zone (Figure 4A). These pathological alterations were paralleled by sustained elevations in plasma total and fractionated bilirubin, as well as increased ALT and AST activities throughout the experimental period (Figure 3H–J).

In the livers of the intact and control animals, heat shock protein-70 (HSP70) was constitutively and uniformly expressed in all hepatocytes, displaying moderate cytoplasmic and nuclear coloration. At 12 h, coincident with the development of necrotic changes in the centrilobular region, a significant decrease in HSP70 expression was observed in the surviving cells within this zone, as evidenced by reduced optical density. A concurrent reduction in HSP70 expression was also noted in the periportal (triad) regions at this time. By 24 h, a well-defined demarcation zone composed of hepatocytes exhibiting high HSP70 expression was clearly established, delineating the boundary between viable and necrotic tissue (Figure 4B,F). In the unaffected centroacinar regions, no significant changes in HSP70 expression were detected.

Concomitantly with these histological alterations, a marked increase in the number of CD45^+^ cells was observed in the livers of APAP-treated mice between 12 and 24 h post-injury, compared to controls. This leukocyte influx was largely attributable to neutrophils and macrophages (Figure 4C,G). In contrast, the hepatic content of CD3^+^ T cells exhibited a significant increase at 6 h, but subsequently fell sharply, decreasing to only occasional cells per high-power field by 24 h (Figure 4D,H).

Assessment of hepatic tissue damage via TUNEL staining revealed a progressive increase in the number of cells with fragmented deoxyribonucleic acid (DNA) across all experimental time points, beginning as early as 6 h. The highest number of TUNEL-positive cells was visualized at 24 h post-injury (Figure 4E,I).

#### 2.6.2. Histological and Functional Characteristics of the Spleen

The development of damage in the liver, the primary target organ, was also accompanied by dystrophic and destructive changes in other organs.

**Figure 5:**
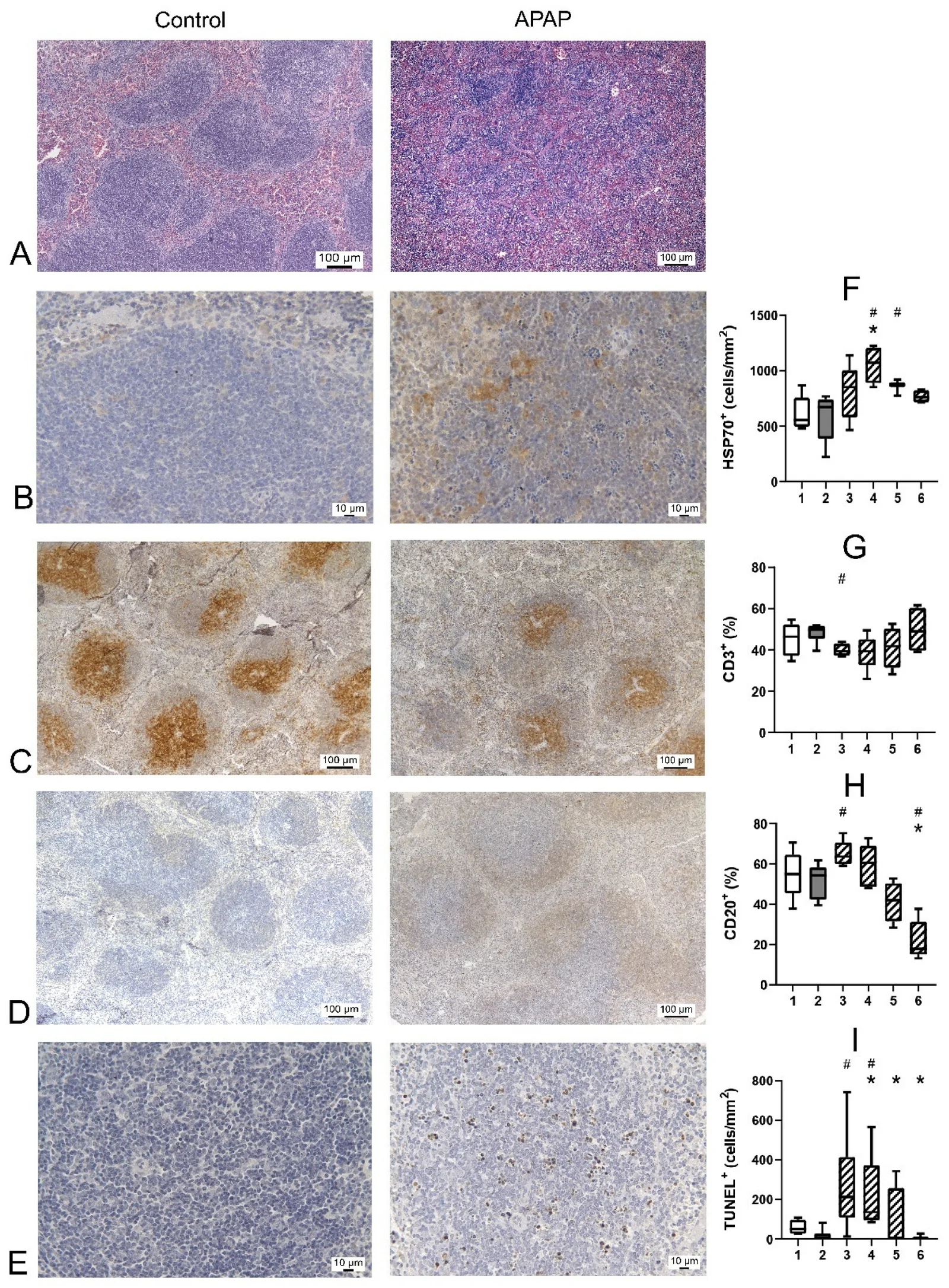
The effect of LD50 acetaminophen (APAP) administration on the spleen histology in C57Bl/6 mice. (**A**) Representative microphotographs of the spleen sections stained by hematoxylin and eosin (H&E). Histoarchitectural disruption and severe white pulp involution are evident. APAP—24 h. Objective magnification 10×. Scale bar—100 µm. (**B**) Representative microphotographs of HSP70 stained spleen sections. APAP—24 h. Objective magnification 40×. Scale bar—10 µm. (**C**) Representative microphotographs of CD3 stained spleen sections. APAP—6 h. Objective magnification 10×. Scale bar—100 µm. (**D**) Representative microphotographs of CD20 stained spleen sections. APAP—6 h. Objective magnification 10×. Scale bar—100 µm. (**E**) Representative microphotographs of TUNEL stained spleen sections. APAP—6 h. Objective magnification 40×. Scale bar—10 µm. (**F**) Spleen HSP70^+^-cells. (**G**) The percentage of the spleen follicle area positive for CD3 staining. (**H**) The percentage of the spleen follicle area positive for CD20 staining. (**I**) Spleen TUNEL^+^-cells. 1—intact group. 2—control group. 3—APAP, 6 h. 4—APAP, 12 h. 5—APAP, 24 h. 6—APAP, 48 h. The data are presented in box and whisker plots: the boxes are drawn from Q1 to Q3, the bold lines show medians, and the whiskers provide the 100% ranges of the values. N = 7 mice per group. Mann–Whitney U-test, *—*p* < 0.05 vs. intact group; #—*p* < 0.05 vs. control group.

Histological evaluation of the spleen from APAP-treated mice revealed evidence of cellular death within the white pulp, as indicated by abundant apoptotic bodies across all time points. From 12 h onward, a substantial subset of animals (4/7) exhibited total loss of red/white pulp demarcation and near-complete lymphoid follicle involution (Figure 5A).

No significant changes in HSP70^+^ cell numbers were noted in the red pulp at any time point. However, the white pulp exhibited a significant elevation in HSP70^+^ cells at 12 h (compared with both controls), sustained through 24 h (compared with the control group), and returning to the levels observed in the intact animals by 48 h (Figure 5B,F).

Immunohistochemically, the white pulp contained discrete CD3^+^ and CD20^+^ cell zones. At 6 h post-injury, the CD3^+^ area was significantly reduced compared with controls, with no significant differences at later time points (Figure 5C,G). Conversely, the CD20^+^ zone significantly expanded at 6 h, subsequently decreasing by 48 h, when significant differences between both comparator groups emerged (Figure 5D,H).

TUNEL staining revealed a marked increase in apoptotic cells within the white pulp at 6 h, sustained through 12 h, followed by a significant decline at 24–48 h relative to the intact controls, with similar trends observed in the red pulp (Figure 5E,I).

#### 2.6.3. Histological and Functional Characteristics of the Kidneys

**Figure 6:**
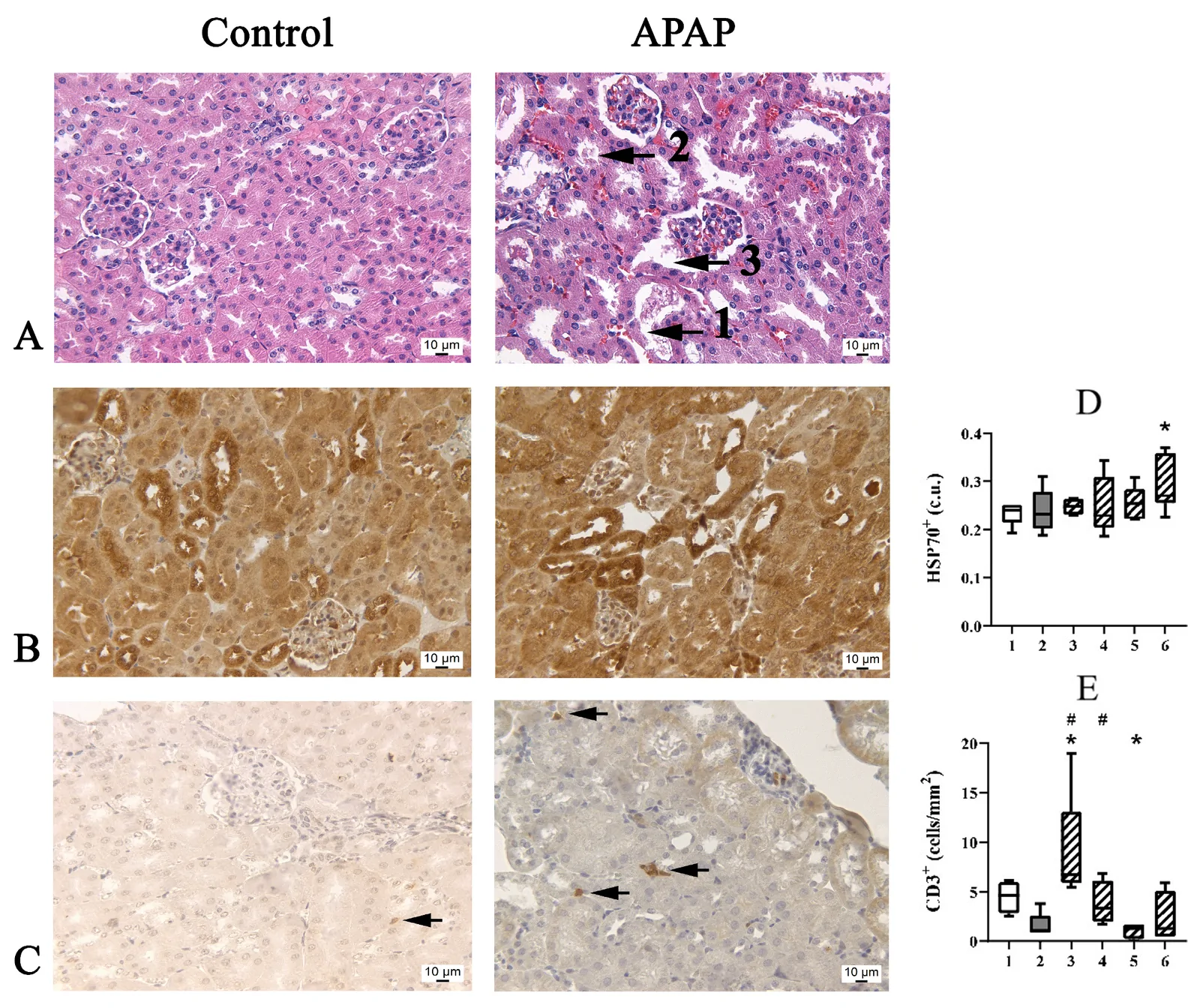
The effect of LD50 acetaminophen (APAP) administration on the renal histology in C57Bl/6 mice. (**A**) Representative microphotographs of the kidney sections stained by hematoxylin and eosin (H&E). 1—brush border destruction. 2—desquamation. 3—expansion of the Bowman capsule space. APAP—48 h. (**B**) Representative microphotographs of HSP70 stained spleen sections. APAP—48 h. (**C**) Representative microphotographs of CD3 stained spleen sections. APAP—6 h. Objective magnification 40×. Scale bar—10 µm. Black arrows denote CD3^+^-cells. (**D**) Optical density of HSP70^+^-cells. (**E**) Kidney CD3^+^-cells. 1—intact group. 2—control group. 3—APAP, 6 h. 4—APAP, 12 h. 5—APAP, 24 h. 6—APAP, 48 h. The data are presented in box and whisker plots: the boxes are drawn from Q1 to Q3, the bold lines show medians, and the whiskers provide the 100% ranges of the values. N = 7 mice per group. Mann–Whitney U-test, *—*p* < 0.05 vs. intact group; #—*p* < 0.05 vs. control group.

Histological examination of the kidneys revealed widening of the Bowman’s capsule space at all experimental time points. Dilatation of the renal tubular lumina was also observed, with some tubules containing sloughed tubular epithelial cells (casts), along with signs of urinary stasis (eosinophilic tubular contents). At 12 h post-administration, evidence of proximal tubular injury emerged, characterized by brush border destruction, cellular disruption, desquamation, and focal eosinophilia and vacuolization of tubular epithelial cells. By 48 h, eosinophilic staining of renal tubules with areas of epithelial degradation was visually apparent, and the lumina of some tubules were filled with eosinophilic material (Figure 6A). Dystrophic changes in the renal glomeruli were also detected.

At that time of the study, the vascular glomeruli were displaying signs of dystrophic changes, and a statistically significant increase in plasma creatinine concentration was observed compared to the intact and control groups (Figure 3K).

HSP70 expression in the kidneys of the C57Bl/6 mice remained stable during the first 24 h post-APAP, subsequently increasing by 48 h relative to the control group in proximal tubules and renal corpuscles, and relative to the intact group in distal tubules (Figure 6B,D).

Quantitative analysis revealed no statistically significant differences in the number of CD45^+^ (leukocytes), CD20^+^ (B-lymphocytes), or TUNEL^+^ cells within the renal tissue at any time point. However, beginning at 6 h following the administration of a semi-lethal dose of APAP, pronounced migration of CD3^+^ cells (T-lymphocytes) into the kidneys was observed, followed by a statistically significant decline in the number of immunopositive cells by 24 h (Figure 6C,E).

#### 2.6.4. Histological and Functional Characteristics of the Lungs

**Figure 7:**
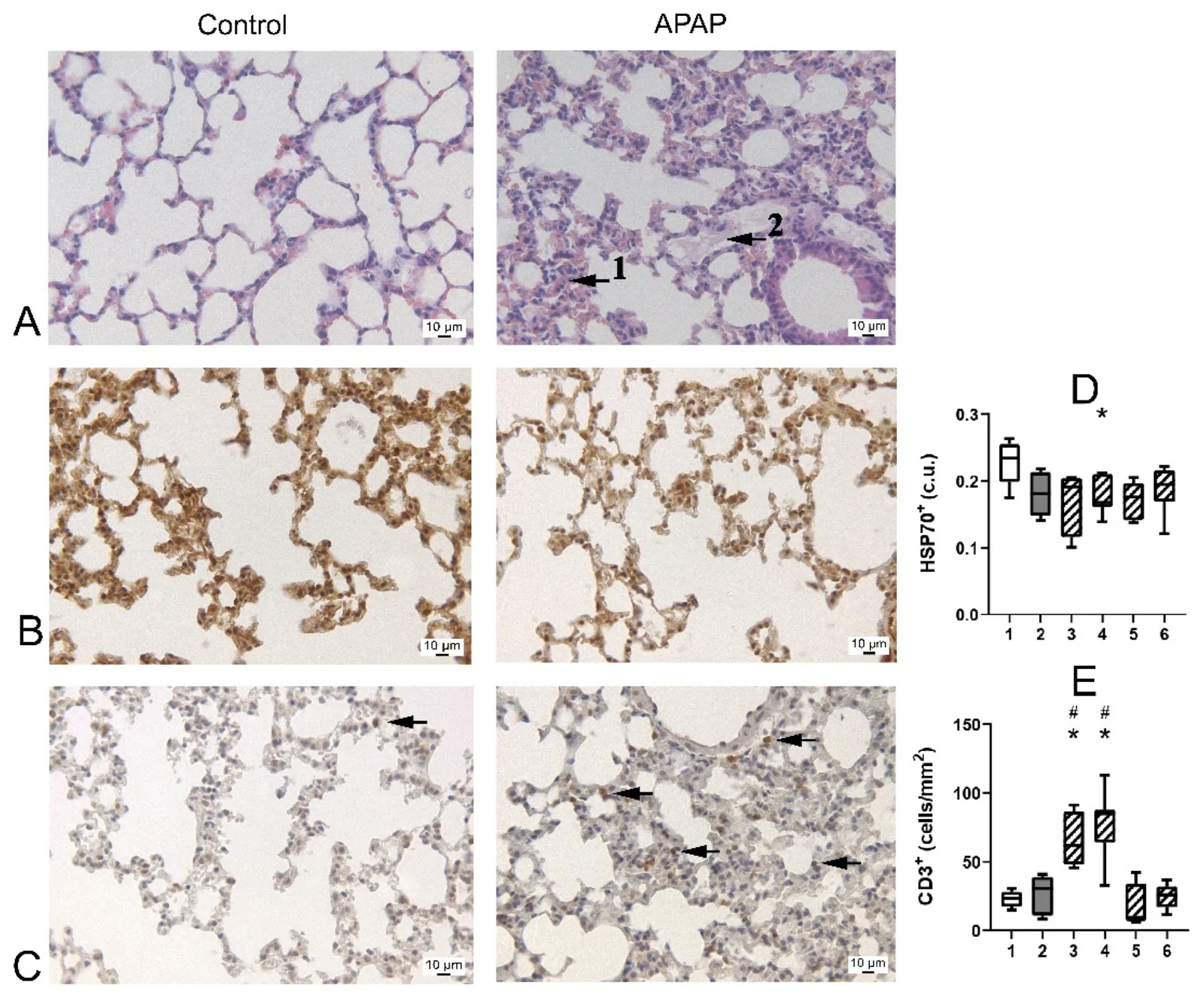
The effect of LD50 acetaminophen (APAP) administration on the lung histology in C57Bl/6 mice. (**A**) Representative microphotographs of the lung sections stained by hematoxylin and eosin (H&E). 1—thickening of the interalveolar septa and alveolar epithelium. 2—exudate in the alveolar lumen. APAP—6 h. (**B**) Representative microphotographs of HSP70 stained lung sections. APAP—6 h. (**C**) Representative microphotographs of CD3 stained lung sections. APAP—6 h. Objective magnification 40×. Scale bar—10 µm. Black arrows denote CD3^+^-cells. (**D**) Optical density of HSP70^+^-cells. (**E**) Lung CD3^+^-cells. 1—intact group. 2—control group. 3—APAP, 6 h. 4—APAP, 12 h. 5—APAP, 24 h. 6—APAP, pronounced leukocyte infiltration in the lungs. *—*p* < 0.05 vs. intact group; #—*p* < 0.05 vs. control group.

At 6 h after APAP administration, histological examination of the lung tissue from experimental animals revealed signs of edema, including thickening of the interalveolar septa and alveolar epithelium, as well as the presence of exudate within the alveolar lumina (Figure 7A). The histological findings are confirmed by a statistically significant increase in the ratio of wet to dry weight at the same time point (intact: 4.29 (4.11–4.39); control: 4.21 (4.00–4.30); 6 h: 4.52 (4.51–5.05)).

HSP70 expression in the lungs did not differ from the comparator groups during the first 6 h post-APAP but decreased by 12 h relative to the intact animals. By 24–48 h, expression levels had returned to baseline (Figure 7B,D).

The number of CD3^+^ T cells in the lungs increased approximately 4-fold by 12 h post-exposure, returning to levels comparable to intact and control groups at 24 and 48 h (Figure 7C,E). Only occasional CD20^+^ (B cells) and TUNEL^+^ cells were observed per high-power field in lung tissue, with no statistically significant differences observed between groups. Likewise, no intergroup differences in CD45^+^ cell count were found, indicating the absence of pronounced leukocyte infiltration in the lungs.

#### 2.6.5. Histological and Functional Characteristics of the Heart

**Figure 8:**
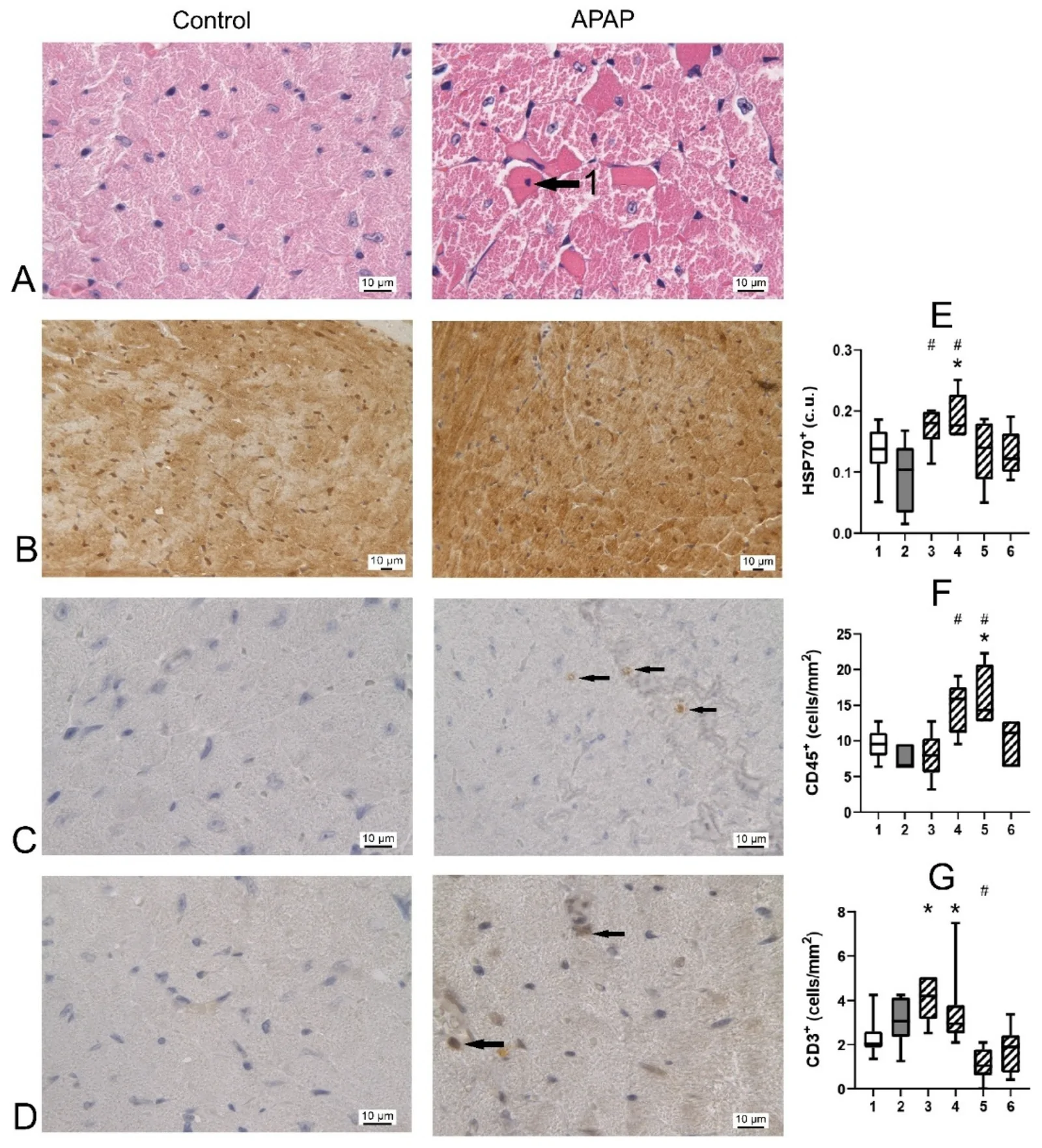
The effect of LD50 acetaminophen (APAP) administration on cardiac histology in C57Bl/6 mice. (**A**) Representative microphotographs of the heart ventricle sections stained by hematoxylin and eosin (H&E). 1—heterogeneous staining of cardiomyocytes with hyperchromatic nuclei. Objective magnification 100×. (**B**) Representative microphotographs of HSP70 stained heart sections. Objective magnification 40×. (**C**) Representative microphotographs of CD45 stained lung sections. APAP—24 h. Objective magnification 100×. Black arrows denote CD45^+^-cells. (**D**) Representative microphotographs of CD3 stained lung sections. APAP—24 h. Objective magnification 100×. Black arrows denote CD3^+^-cells. (**E**) Optical density of HSP70^+^-cells. (**F**) Heart CD45^+^-cells. (**G**) Heart CD3^+^-cells. Scale bar—10 µm. 1—intact group. 2—control group. 3—APAP, 6 h. 4—APAP, 12 h. 5—APAP, 24 h. 6—APAP, 48 h. The data are presented in box and whisker plots: the boxes are drawn from Q1 to Q3, the bold lines show medians, and the whiskers provide the 100% ranges of the values. N = 7 mice per group. Mann–Whitney U-test, *—*p* < 0.05 vs. intact group; #—*p* < 0.05 vs. control group.

At early time points (6–12 h post-exposure), no histological alterations in cardiac tissue were observed. Beginning at 24 h after APAP administration, signs of dystrophic changes became evident in the myocardium, including heterogeneous staining of cardiomyocytes with hyperchromatic nuclei and loss of transverse striations (Figure 8A).

A statistically significant increase in the plasma troponin I concentrations of the experimental animals compared to the controls was observed at all time points of the experiment (Figure 3L).

HSP70 expression in transverse sections of cardiac muscle remained at levels comparable to the control groups during the first 24 h post-toxicant administration, with an increase relative to the control group observed by 48 h. In longitudinal sections, increased HSP70 immunoreactivity was already evident at 6 h, maintaining statistically significant differences from the comparator groups through 12 h. By 24–48 h, expression had declined to baseline levels (Figure 8B,E).

Total leukocyte counts (CD45-expressing cells) were elevated in the experimental animals at 12 h compared to the control group (Figure 8C,F). Only single CD20^+^ (B-lymphocytes) and TUNEL^+^ cells were detected per high-power field in cardiac tissue, with no statistically significant intergroup differences. In the heart, the number of T-lymphocytes (CD3-expressing cells) increased at 12 h post-toxicant administration relative to the intact group and subsequently decreased by 24 h relative to the control group (Figure 8D,G).

## 3. Discussion

Although various aspects of paracetamol poisoning pathogenesis have been studied extensively, the pathophysiological relevance of sterile inflammation in APAP toxicity remains controversial [10,11]. We hypothesized that SI may represent the core pathogenic mechanism in severe APAP overdose, driving secondary damage.

Our earlier clinical work has identified prototypical signs of SI in a range of critical conditions, including aseptic processes such as acute trauma, massive obstetric hemorrhage, amniotic fluid embolism, and hemorrhagic stroke [31,32,33]. In all these cases, SI onset was consistently associated with shock, DIC, multiorgan dysfunction, and a substantially increased risk of death.

In this work, we examined the main SI components in male C57Bl/6 mice after a semi-lethal acetaminophen dose, including SIR, tissue injury, MCD, MOF, and HPA axis activation [12].

The pathophysiology of APAP toxicity is broadly similar in mice and humans [28]. This makes the APAP-induced mouse model of ALF highly representative for studying the human condition and allows detailed morphological and functional analysis. Experimental models offer precise dose control, standardized time courses, and the ability to correlate various pathological changes—advantages that are particularly valuable in APAP poisoning, because clinical therapy may mask or even prevent the development of SI.

Admittedly, the standard murine APAP model uses a 12 h fast, which sensitizes the liver to injury via glutathione depletion, CYP induction, and impaired detoxification [34,35]. While such prolonged fasting is uncommon in humans before an APAP overdose, fasting does potentiate APAP toxicity in humans as well [36]. Therefore, this methodological detail does not materially affect the model’s utility for studying APAP pathogenesis.

Acute APAP overdose causes massive hepatocellular necrosis and releases DAMPs into the circulation. These DAMPs—including HMGB1, histones, DNA fragments, and other intracellular components—activate immune cells [37,38]. Monocytes and macrophages recognize them and begin producing pro-inflammatory cytokines and chemokines. Once in the bloodstream, these mediators drive a cytokine storm, i.e., a SIR. This storm activates macrophages, neutrophils, dendritic cells, and vascular endothelial cells. It also stimulates antigen-presenting cells, which further increase cytokine production, creating a self-perpetuating vicious cycle [39,40].

The SIR—and in particular the cytokine storm—is a key driver of SI. Our data show that acute APAP-induced liver injury in C57Bl/6 mice triggered a strong SIR, reflected by a substantial multi-fold increase in cytokines (IL-1α, IL-6, IL-10, and TNF-α) and acute-phase reactants (CRP).

A range of experimental models are used to study SI, including the lipopolysaccharide-induced sepsis model. Acute liver failure appears to share mechanistic features with septic shock in terms of SI and multiple organ dysfunction [41]. Our findings support this: the levels of pro-inflammatory (TNF-α, IL-6) and anti-inflammatory (IL-10) cytokines in C57Bl/6 mice were comparable to, or even markedly higher than, those reported in the LPS-induced sepsis model using the same mouse strain [42].

In contrast to our data, Nithiyanandam and Prince [25] observed that APAP-induced intoxication in Wistar rats leads to a 2- to 4-fold elevation of systemic TNF-α and IL-6 and a 4-fold reduction in IL-10. The observed discrepancies probably reflect species-dependent variations in APAP metabolism. It is widely acknowledged that rats are less appropriate for this type of investigation, whereas the mouse model closely mirrors the human pathophysiology of paracetamol overdose [9].

Our data on IL-10 elevation are consistent with Roth et al. [26], who reported a significant increase in IL-10 in mice given 600 mg/kg APAP at 24 h. However, by monitoring earlier time points, we detected the rise as early as 6 h. Roth et al. further demonstrated that excessive IL-10 production impairs monocyte recruitment to the injured liver and suppresses fibrinolysis, thereby delaying necrotic debris clearance and liver regeneration, ultimately leading to unfavorable outcomes.

Woolbright et al. [23] assessed cytokine dynamics in APAP-overdose patients and found that, regardless of outcome, those with ALF had modest increases in pro-inflammatory cytokines (IL-1α, IL-1β, TNF-α). In contrast, non-survivors showed significantly higher IL-6 and IL-10 than survivors. The authors suggested that high day-1 IL-10 may serve as a poor prognostic marker. Differences between our findings and theirs may be attributable to variations in disease severity and the relatively small sample sizes.

Thus, no consensus has yet been reached regarding the dynamics of the main cytokines or their role in APAP overdose pathogenesis. In a review, Iracheta-Vellve and Szabo [10] highlighted the importance of IL-1α in APAP-induced liver failure, but noted its dual function as both an alarmin and a cytokine. They also pointed out that it remains unclear whether circulating IL-1α is actively secreted, or passively released by Kupffer cells as a DAMP, and which of these roles contributes more significantly to the disease process. The apparent discrepancies between the findings of different studies are likely attributable to variations in clinical or experimental conditions. Moreover, the role of individual cytokines may depend on the phase of the pathological process and their prevailing levels. Importantly, many researchers view inflammation in APAP-induced liver injury as a pro-resolving, regenerative event, rather than attributing a destructive role to cytokines. Our study, however, clearly demonstrates the detrimental aspects of inflammation in APAP overdose. Moreover, there is still no consensus on the dynamics of the measured parameters in severe APAP poisoning, particularly at early time points. This highlights the value of our work.

A hallmark of the transition from local to systemic inflammation is systemic alteration, which varies in intensity. It involves both primary and secondary tissue damage, accompanied by dystrophic changes in organs [43]. This phenomenon can be detected either by identifying the alteration factors themselves or by assessing their consequences, reflected in circulating levels of tissue breakdown products [12].

MOF, a cardinal feature of SI, is also known to accompany APAP-induced liver injury and worsen its prognosis [17]. Importantly, MOF does not always reflect parenchymal cell death; it may also involve dysfunction or depletion of tissue-resident immune cells, driven by a compensatory anti-inflammatory response [44].

Therefore, studying SI in APAP-induced hepatotoxicity should include not only morphological changes in organs but also immune cell migration. We used immunohistochemistry to quantify total leukocytes, T-lymphocytes, and B-lymphocytes (based on CD45, CD3, and CD20 expression, respectively) in organs after APAP injury. To assess cell death that may contribute to MOF, we performed TUNEL staining to detect fragmented DNA in C57Bl/6 mice.

Cells often respond to stress by upregulating heat shock proteins. HSP70 exerts cytoprotective effects under various pathological conditions by binding to intracellular proteins to restore their native conformation or facilitating their elimination [45]. We therefore examined HSP70 expression in organ tissues, focusing on its potential protective role during acute APAP intoxication.

In the liver, the main target organ, hepatocyte dystrophy was already apparent at 6 h post-APAP. Necrotic foci then developed in the centrilobular areas, delineated by a clear inflammatory rim. Functionally, these changes translated into elevated bilirubin and transaminase activities from 6 h onward, with ALT predominating over AST—a pattern consistent with the early injury phase [46].

The spleen also underwent destructive and involutive changes, especially in the white pulp, where apoptotic bodies accumulated in lymphoid follicles and the normal red/white pulp architecture was disrupted.

Renal injury was also evident: tubular epithelium was affected early, with congestion, and glomerular involvement emerged by 48 h. The >1.5-fold creatinine rise at this time point is considered by several authors to indicate acute renal failure [47,48].

In our study, pulmonary changes included edema and alveolar exudate at 6 h. The progression from interstitial to alveolar edema is typical of acute lung injury and subsequent ARDS—a classic life-threatening complication of SI, regardless of its etiology [49].

Plasma troponin I, a specific marker of cardiac injury, was raised throughout the experiment. Its release can be reversible (via transient membrane leakage) or irreversible (following cell death). In the absence of infarction, this rise likely signals systemic alteration and poor prognosis [50]. Cardiac histological changes emerged at 24 h post-APAP, confirming secondary myocardial injury. Our results corroborate the marker’s utility in mice, as previously shown in patients with acute trauma [31].

Critical thrombocytopenia (platelet count < 450 × 10^3^/μL) has been reported as a marker of systemic microthrombosis [51]. In the present study, we observed that a semi-lethal dose of APAP in C57Bl/6 mice elicited thrombocytopenia from 12 h onwards, with a peak at 24 h. These findings are suggestive of impaired coagulation homeostasis in our experimental setting.

**Figure 9:**
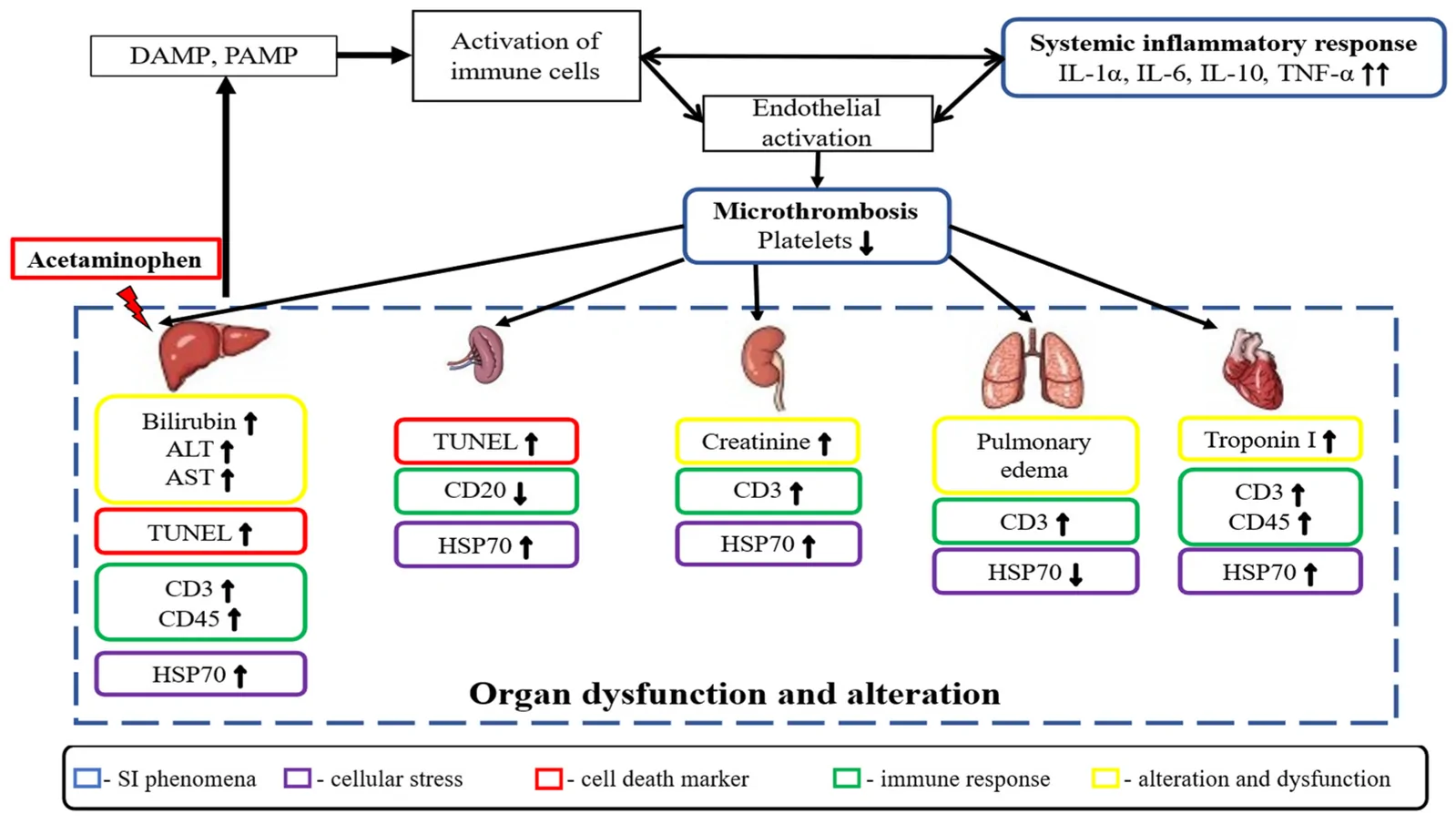
Systemic inflammation (SI) in life-threatening acetaminophen (APAP) poisoning: a proposed mechanism. DAMP—damage-associated molecular patterns. PAMP—pathogen-associated molecular patterns. IL—interleukin. TNF-α—tumor necrosis factor. ALT—alanine aminotransferase. AST—aspartate aminotransferase. HSP70—heat shock proteins-70. The colored frames indicate our experimental data; the black frames indicate data from the literature.

Figure 9 provides a schematic overview of the proposed systemic inflammatory cascade triggered by APAP-induced liver injury, integrating the SI phenomena described in the literature with those observed in our study.

Primary APAP-induced liver injury, evidenced by elevated bilirubin, transaminases, and hepatocellular necrosis, likely triggered DAMP release into the circulation. According to the literature, this initiated immune cell activation and a sharp rise in cytokine production (SIR). Hypercytokinemia, together with the systemic effects of damaging factors, promoted microcirculatory disturbances (thrombocytopenia), cellular stress and death, systemic alteration, and immune cell recruitment to the liver, heart, lungs, and kidneys. Ultimately, these SI phenomena culminated in secondary organ damage and progressive multiple organ dysfunction.

In summary, the present study identified four key features of SI in severe APAP-induced poisoning: SIR, MCD, systemic alteration, and MOF.

Several limitations should be acknowledged. First, the APAP dosage used in our study is specific to the experimental conditions (mouse strain, sex, age, fasting duration, route of administration, etc.). The translational potential of the dose employed from the murine model to humans is constrained by considerable interspecies variations in APAP metabolism. The 12 h fasting protocol employed depletes endogenous glutathione stores, which may potentiate APAP-induced injury. Since patients with APAP overdose rarely fast for such prolonged periods, the manifestation of SI in humans may occur at higher ingested doses. Furthermore, the intraperitoneal route used in the present study differs from the oral route, which is the predominant mode of APAP exposure in humans, and this discrepancy may also influence dose-dependent effects.

Second, our pilot study was confined to a limited set of biomarkers for each specific aspect of SI, namely CRP to evaluate the acute-phase response, platelet counts to assess microthrombosis, and corticosterone to measure HPA axis activation. Although we found elevated plasma IL-1α and hepatic HSP70 (both recognized as DAMPs) and confirmed hepatocyte necrosis by histology and biochemistry, plasma total DAMPs were not directly measured. The inclusion of a broader range of markers is certainly critical and constitutes a major goal of our continuing studies.

Third, our corticosterone data indicate that intraperitoneal injection may trigger a non-specific HPA axis response. Consequently, corticosterone is of limited utility as a surrogate marker for HPA activation in this SI model. Future studies should employ less invasive administration routes or more specific neuroendocrine biomarkers.

## 4. Materials and Methods

### 4.1. Animals and Ethical Approval

Ten-week-old male C57Bl/6 mice were used in this study. All animal procedures were performed in the vivarium of the Institute of Immunology and Physiology of the Ural Branch of the Russian Academy of Sciences (IIP UB RAS), in accordance with the European (EU Directive 2010/63/EU, DL 26/2014) and national (GOST 33216–2014) regulations. All experimental protocols were approved by the Ethics Committee of the Institute of Immunology and Physiology, Ural Branch of the Russian Academy of Sciences (protocol No. 04/22, 18 November 2022). The animals were maintained under standard conditions (12 h light/dark cycle, 22 ± 2 °C, ad libitum access to food and water) throughout the study.

### 4.2. Experimental Design

The animals were randomly assigned to six groups of seven mice each: naive, control, and four experimental groups. After a 12 h fast [34], the experimental mice received a single intraperitoneal injection of APAP (Sigma-Aldrich, 14 mg/mL in saline) at 600 mg/kg, the LD50 for this strain, sex, and age, as determined in pilot experiments [52]. The controls received an equal volume of saline. The selection of the LD50 dose was motivated by prior evidence that systemic inflammation correlates with roughly 50% mortality in a range of human pathologies [31]. To minimize animal distress, humane endpoints were predefined based on clinical signs characteristic of a moribund state. The euthanasia criteria included: (i) a decrease in body weight exceeding 20% relative to baseline; (ii) unresponsiveness to external stimuli; and (iii) overt manifestations of severe and persistent pain, as evaluated by the facial grimace scale and whisker posture. For the first 3 h following APAP administration, the animals were kept under continuous observation. Thereafter, monitoring was performed at intervals not exceeding 12 h. Whenever the predefined endpoints were met, the animals were euthanized without delay. No deaths occurred within 12 h, but mortality at 24 and 48 h gave an overall 70% survival rate. Post-mortem changes precluded sampling from dead animals; therefore, sample sizes reported correspond only to surviving animals from which viable tissues and blood were collected at scheduled endpoints.

**Figure 10:**
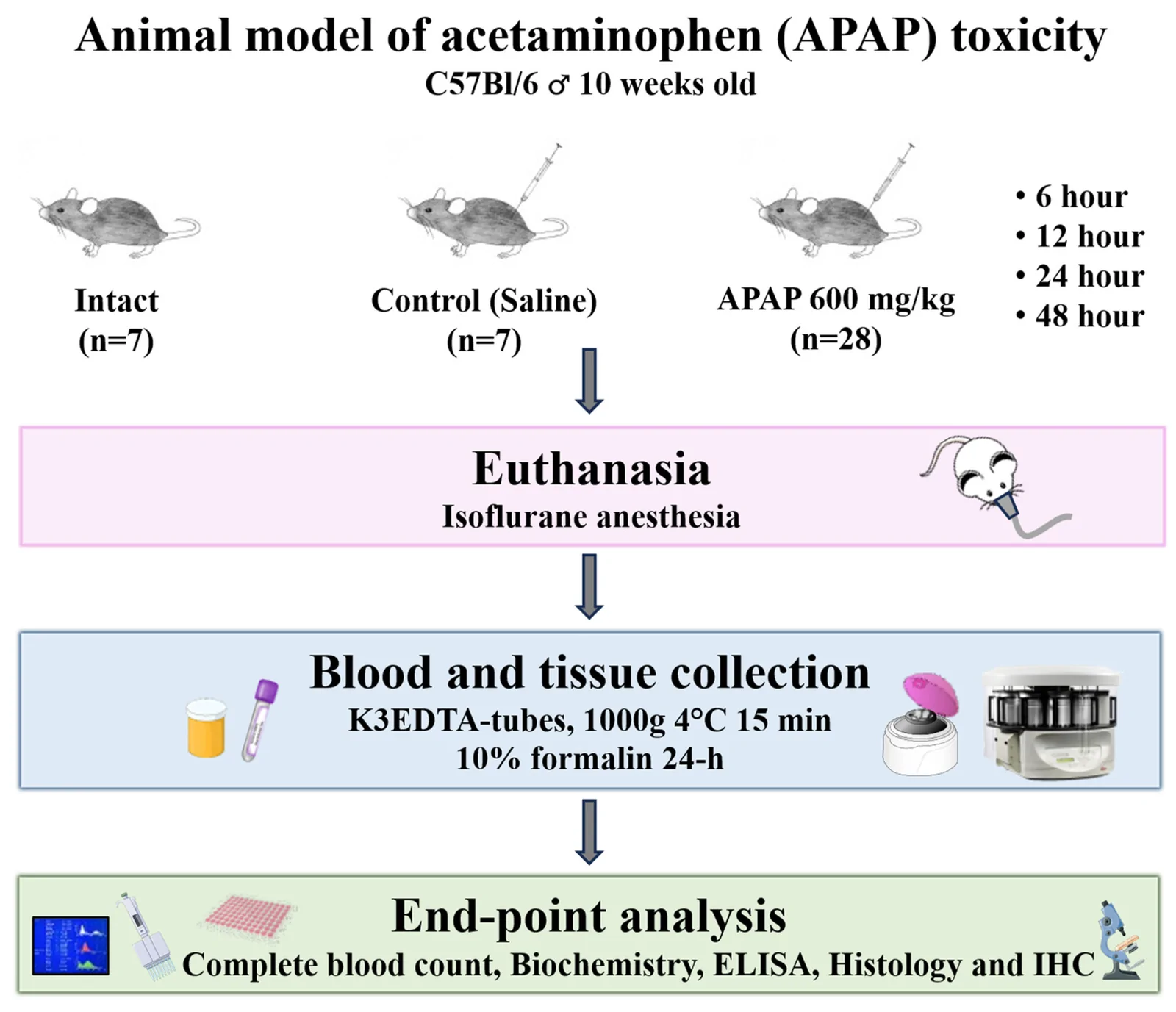
The experimental design and study protocol. The mice were divided into three groups: intact (untreated), control (saline), and APAP (600 mg/kg). At the indicated time points, blood and tissues were collected to evaluate hematological and plasma biochemical parameters, cytokine levels (ELISA), as well as organ histology and immunohistochemistry (IHC). n = 7 mice per group per time point were used for all assessments.

The experimental animals were euthanized at 6, 12, 24, and 48 h after injection (the controls at 24 h). All mice were deeply anesthetized with isoflurane (Laboratories Karizoo, Spain) and subsequently euthanized by exsanguination at the respective time points. A schematic representation of the study design is shown in Figure 10.

### 4.3. Clinical Severity Score and Physiological Monitoring

All mice were continuously monitored for clinical status using a CSS [29]. that assessed physical appearance and behavior (e.g., piloerection, hunched posture, lethargy, reduced locomotion). Body weight was recorded before treatment and at each endpoint. Food intake, water consumption, respiratory rate (based on chest wall movements), and urine output were all evaluated qualitatively by visual inspection.

### 4.4. Sample Collection

Blood was collected from the femoral vein into K_3_-EDTA-coated Microvette tubes (Sarstedt, Germany) (0.2 mL). Plasma was obtained by centrifugation at 1000× *g* for 15 min at 4 °C (Sigma 3K30; Sigma Laborzentrifugen GmbH, Osterode am Harz, Germany), aliquoted into 100-µL portions, snap-frozen, and stored at −80 °C until analysis. For histological and immunohistochemical examinations, internal organs (liver, kidneys, spleen, lungs, and heart) were harvested and fixed in 10% buffered formalin.

### 4.5. Hematological Analysis

Hematological parameters were determined using a Mindray BC-2800Vet analyzer (Mindray, Shenzhen, China). The neutrophil-to-lymphocyte ratio (NLR) was then calculated as an indicator of systemic inflammation and stress in critical conditions [53].

### 4.6. Determination of Systemic Inflammatory Response Indicators

The concentrations of interleukin (IL)-1α (CSB-E04621m, Cusabio, Houston, TX, USA), IL-6 (CSB-E04639m-IS, Cusabio), IL-10 (CSB-E04594m, Cusabio), tumor necrosis factor (TNF)-α (CSB-E04741m-IS, Cusabio), and C-reactive protein (CRP; RK09103, ABclonal Biotechnology Co., Ltd.) in blood plasma were measured using ELISA. All assays were performed on the Lazurite Automated ELISA System (Dynex Technologies Inc., Chantilly, VA, USA) according to the manufacturers’ protocols.

Based on preliminary hematological and biochemical analyses showing no significant differences among saline-treated groups across all time points (6, 12, 24, and 48 h), we collected control samples for ELISA only at 24 h. This approach minimized animal use, in line with bioethical standards and the local ethics committee’s recommendation.

### 4.7. Determination of the Microthrombosis Index

Absolute platelet counts, used as a criterion for paracoagulation in acute critical conditions, were determined using a Mindray BC-2800Vet hematology analyzer (Mindray, China).

### 4.8. Evaluation of HPA Axis Activation

Plasma corticosterone levels were measured by ELISA using a commercial kit (CSB-E07969m, Cusabio).

### 4.9. Assessment of Multiple Organ Dysfunction and Morphological Alterations

Troponin I concentration was determined by ELISA (RK09274, ABclonal Biotechnology Co., Ltd., Woburn, MA, USA). Plasma ALT and AST activities, as markers of tissue alteration, as well as total bilirubin and creatinine levels, as markers of liver and kidney dysfunction, respectively, were measured using standard biochemical reagent kits from Vital Diagnostics (St. Petersburg, Russia).

For histopathological evaluation, internal organs were fixed in 10% neutral buffered formalin, washed in cold tap water, and processed through a standard dehydration and clearing series using a Leica TP 1020 automated tissue processor (Leica Microsystems, Wetzlar, Germany). Tissue fragments were embedded in paraffin and mounted on standard histological cassettes using a Leica EG 1160 automatic embedding system (Leica Microsystems, Germany). Sections of 3–5 μm thickness were cut on a Leica SM 2000R microtome (Leica Microsystems, Germany) and stained with haematoxylin and eosin using a Leica ST 5010 automated staining system (Leica Microsystems, Germany). Histological images were acquired with a Leica DM2500 light microscope (Leica Microsystems, Germany) equipped with a Leica DFC420 digital camera (Leica Microsystems, Germany).

The severity of pulmonary edema was assessed by the wet-to-dry weight (W/D) ratio using a gravimetric method [54].

The immunohistochemical staining protocol for CD3 (C7930, Sigma-Aldrich, St. Louis, MO, USA), CD20 (PA5–16701, Thermo Fisher Scientific, Waltham, MA, USA), CD45 (ab281586, Abcam, Cambridge, UK) and HSP70 (sc-32239, Santa Cruz Biotechnology, Dallas, TX, USA) comprised the following steps: deparaffinization according to the standard protocol using an automated histological staining system (Leica ST5010, Leica Microsystems, Germany); antigen retrieval using citrate buffer; blockade of endogenous peroxidase activity with 3% hydrogen peroxide; blocking of non-specific staining in tissues with high endogenous biotin content using an avidin–biotin blocking kit; incubation with primary and then secondary antibodies; incubation with horseradish peroxidase; incubation with 3,3′-diaminobenzidine; counterstaining with hematoxylin; dehydration in alcohols; and clearing in xylene using the automated Leica ST5010 staining system (Leica Microsystems, Germany), followed by mounting in Canada balsam.

Positively stained cells were counted in 20 fields of view at ×1000 magnification for CD20^+^ and CD45^+^ cells, and at ×400 magnification for CD3^+^ cells, with subsequent recalculation of cell numbers per 1 mm^2^ of section area.

The relative area of white pulp zones in the spleen expressing CD3 or CD20 was determined using FIJI ImageJ 1.52 software (National Institutes of Health, Bethesda, MD, USA). Calculations were performed on no fewer than 10 fields of view.

Assessment of HSP70 functional activity in cells was performed by measuring optical density in relative units using Videotest-Morphology 5.2 software (VideoTest, St. Petersburg, Russia).

Cells with fragmented DNA were detected using the Elabscience TUNEL In Situ Apoptosis Kit (HRP-DAB method) (Elabscience, Houston, TX, USA) according to the manufacturer’s protocol. The number of positively stained cells was counted at ×400 magnification and subsequently recalculated per 1 mm^2^ of section area.

### 4.10. Statistical Analysis

Statistical analysis was performed using Statistica 12.0 (StatSoft, Inc., Tulsa, OK, USA). The Shapiro–Wilk test was used to assess the normality of the distribution. Since the data were not normally distributed, they are presented as the median (Me) with 25th and 75th percentiles (Q1–Q3), and the whiskers correspond to the minimum and maximum values. Comparisons between groups were performed using the Mann–Whitney U test. A *p*-value of <0.05 was considered statistically significant.

## 5. Conclusions

Severe APAP-induced liver injury in C57Bl/6 mice elicits a critical SIR, paracoagulation, systemic alteration, and tissue destruction. Our findings clearly indicate that SI contributes to the pathogenesis of paracetamol poisoning and poor outcome. Future studies should focus on rigorous model validation and confirmation of the proposed mechanisms.

## Data Availability

The original contributions presented in this study are included in the article. Further inquiries can be directed to the corresponding author.

## Abbreviations

The following abbreviations are used in this manuscript:

APAP	acetaminophen
ALF	acute liver failure
ALT	alanine aminotransferase
ARDS	acute respiratory distress syndrome
AST	aspartate aminotransferase
CRP	C-reactive protein
CSS	clinical severity score
DAMPs	damage-associated molecular patterns
DIC	disseminated intravascular coagulation
DNA	deoxyribonucleic acid
ELISA	enzyme-linked immunosorbent assay
HPA	hypothalamic–pituitary–adrenal system
HSP70	heat shock protein-70
LD50	lethal dose, 50%
MCD	microcirculatory disorders
MOF	multiple organ failure
NAPQI	N-acetyl-p-benzoquinone imine
PAPS	3′-phosphoadenosine-5′-phosphosulfate
ROS	reactive oxygen species
SI	systemic inflammation
SIR	systemic inflammatory response
SOFA	Sequential Organ Failure Assessment scale
TUNEL	Terminal deoxynUcleotidyl transferase Nick-End Labeling
